# The Impact of COVID-19 on Sleep in Autistic Adults: Longitudinal Comparisons pre and During Lockdown

**DOI:** 10.3389/fpsyt.2021.708339

**Published:** 2021-09-21

**Authors:** Elizabeth J. Halstead, Emma C. Sullivan, Dagmara Dimitriou

**Affiliations:** ^1^Sleep Education and Research Laboratory, Psychology & Human Development Department, University College London Institute of Education, London, United Kingdom; ^2^The National Institute for Stress, Anxiety, Depression and Behavioural Change (NISAD), Helsingborg, Sweden

**Keywords:** sleep, sleep quality, pre-sleep arousal, autism, pandemic, COVID-19, adults

## Abstract

**Background:** The longer-term impact of the pandemic on autistic adults' sleep are yet to be revealed, with studies concentrating on sleep in autistic children or mental health outcomes and coping strategies of autistic adults. Given the prevalence of sleep problems experienced by autistic adults and the changes in routine that have occurred as a result of COVID-19 societal restrictions, this study assessed the impact of the COVID-19 pandemic on sleep problems via a longitudinal subjective assessment method.

**Methods:** Sleep data were gathered at three time points from 95 autistic adults, namely prior to the pandemic, at the start of COVID-19 and several months into COVID-19 to obtain a rich longitudinal dataset ascertaining how/if sleep patterns have changed in autistic adults over these several months.

**Results:** In comparison to pre-lockdown, several sleep components were shown to improve during the lockdown. These improvements included reduced sleep latency (time taken to fall asleep), longer sleep duration, improved sleep efficiency, improved sleep quality, as well as improved daytime functioning. Pre-sleep cognitive arousal scores were found to decrease compared to pre-lockdown, meaning cognitive arousal improved. Approximately 65% of participants reported that they felt their sleep had been impacted since COVID-19 since Time 1, with the most common reasons reported as waking up exhausted (36.92%), not being able to get to sleep (33.85%), waking up in the night (29.23%), having a disrupted sleep pattern (27.69%), and nightmares (18.46%).

**Conclusions:** Improvements in sleep may be related to societal changes (e.g., working from home) during the pandemic. Some of these changes are arguably beneficial for autistic adults in creating a more autism-inclusive society, for example telehealth opportunities for care. Further exploration of the associations between mental health and sleep are warranted.

## Introduction

Autism is defined as a permanent developmental condition marked by difficulties in social communication, restricted interests and sensory processing (1). An accumulating body of research has suggested sleep problems are some of the most common co-occurring problems experienced by autistic individuals across their lifespan (2). Autistic adults have been found to have greater sleep problems such as lower sleep quality, longer sleep onset latency and lower sleep efficiency when assessed both objectively (e.g., via actigraphy) and subjectively (e.g., via self-reported questionnaires), compared to typically developing individuals [e.g., (3)]. Given the high prevalence of sleep problems in autistic adults, daily functioning and quality of life may be disproportionally impacted. A recent meta-analysis found autistic adults were significantly more impaired in six out of eleven subjective sleep parameters obtained from questionnaires and sleep diaries (4). Compared to controls, autistic adults showed: higher global Pittsburgh sleep quality index (PSQI) score, higher wake after sleep onset (in minutes and number of awakenings), longer sleep onset latency, longer time spent napping and lower sleep efficiency. Furthermore, autistic adults were also significantly more impaired in ten out of seventeen objective parameters obtained from actigraphy and polysomnography. Again, compared to controls, autistic adults had higher sleep fragmentation and wake after sleep onset (minutes), and a longer total time spent in bed and longer sleep latency onset. Sleep efficiency was significantly lower in autistic adults compared to control participants. The authors concluded that autistic adults had impaired sleep when compared to controls across a range of subjective and objective sleep parameters.

The global COVID-19 pandemic, caused by the SARS-CoV-2 coronavirus, has brought uncertainty to the world, and has impacted on how we lead our day-to-day lives. Various COVID-19 related measures and rules have forced us to make rapid changes in our lives and societies worldwide. It is thus likely that that the pandemic itself and related changes have an impact on mental health, with some individuals experiencing more acute effects than others do. Emerging evidence has demonstrated autistic adolescents and children display altered circadian function and behaviour as a result of the pandemic (5–7). Furthermore, in comparison to families with typically developing children, families with autistic children reported greater behavioural problems and elevated parental distress during the resultant pandemic lockdown in an Italian sample (6). Autistic adults may also be particularly vulnerable to the impact of the COVID-19 pandemic. Recent findings demonstrate mental health has deteriorated as a result of the pandemic, evident through increased anxiety and depression and greater stress levels (8, 9). Also, it has been suggested that susceptibility to viral infections may be more prevalent in autistic adults due to health vulnerabilities such as co-occurring complex physical and mental conditions and melatonin deficiencies (10–13).

In addition, a common characteristic of autism is a preference for consistency and adherence to routine, these are two factors have been impacted greatly by the pandemic. This is supported by evidence that adjustment to changes in routine, worries about family and friends, accessing medication, food and personal safety, and general uncertainty about the future have impacted mental health and well-being negatively in autistic adults (8, 14, 15). Moreover, autistic adults may be affected to varying degrees by the pandemic. For autistic adults, the following factors have differentially influenced individual impacts of the pandemic, including; being female, being younger, having a mental health diagnosis prior to the pandemic, or knowing someone who has shown symptoms or has been infected by the virus. Autistic adults exposed to one or more of these risk factors reported more areas negatively impacted by the pandemic (e.g., school, social life, employment) and greater difficulty in coping with these impacts (9). Taken together, evidence suggests that autistic adults may be particularly vulnerable to the impact of the pandemic albeit with varying severity.

The longer-term impact of the pandemic on autistic adults' sleep are yet to be revealed, with studies concentrating on sleep in autistic children or mental health outcomes and coping strategies of autistic adults. In the general population, findings demonstrate a reduction in sleep quality as a result of the COVID-19 pandemic and the resultant restrictions imposed; this is indexed through evidence of short sleep duration and disrupted sleep-wake times in comparison to pre-pandemic sleep quality (16–20). Given the high prevalence of sleep problems experienced by autistic adults, and the changes in routine driven by the COVID-19 societal restrictions, this study assessed the impact of the COVID-19 pandemic on sleep problems via a longitudinal subjective assessment method. Sleep data were gathered at three time points, namely prior to the pandemic, at the start of the COVID-19 pandemic and ~3 months into the COVID-19 pandemic to obtain a rich longitudinal dataset ascertaining how/if sleep patterns have changed in autistic adults over this time period. It was hypothesised, if indeed autistic adults have adjusted well to the societal measures implemented, sleep problems will have improved during the pandemic period in comparison to the pre-pandemic period. However, given the scarcity of research during this time period, our study takes an exploratory approach.

## Materials and Methods

### Participants

A total of 95 autistic adults completed the survey at all three time points (22 males, 59 females, 13 non-binary, 1 preferred not to say). Participants were recruited for Time 1 via social media and autism support groups. At Time 2 and Time 3, participants who agreed to be contacted for future studies were invited to take part via email. Participants were aged between 18 and 65 years old (*M* = 36.86, *SD* = 11.26). Demographic information collected at Time 1 is displayed in Table 1 including socioeconomic related variables (employment, education level and index of multiple deprivation decile) and ethnicity. All participants identified as an autistic adult [81 were diagnosed by a healthcare professional (HCP), 14 were self-diagnosed]. 12.6% of the total sample (*n* = 12) reported no pre-existing health issues prior to the COVID-19 pandemic.

**Table 1:** Summary of autistic adults' demographic information collected at Time 1.

**Demographic variables**	***N***	**%**
**Index of multiple deprivation decile**		
1 – Most deprived	5	5.3
2	3	3.2
3	6	6.3
4	5	5.3
5	8	8.4
6	5	5.3
7	6	6.3
8	1	1.1
9	4	4.2
10- Least deprived	3	3.2
Unknown/Not Reported	49	51.6
**Education level**		
Secondary school up to 16 years	7	7.4
Higher, secondary or further education	25	26.3
Undergraduate degree	34	35.8
Postgraduate degree	28	29.5
Unknown/Not Reported	1	1.1
**Ethnicity**		
White	84	88.4
Black African	1	1.1
Black Caribbean	1	1.1
Hispanic	1	1.1
Romani	1	1.1
Latina	1	1.1
Mixed ethnicity	3	3.2
Unknown/Not reported	3	3.2
**Employment status**		
Employed (full time)	25	26.3
Employed (part time)	14	14.7
Self employed	7	7.4
Student	20	21.1
Retired	2	2.1
Unemployed looking for work	3	3.2
Unemployed not looking for work/unable to work	5	5.3
On disability allowance	15	15.8
Home maker	3	3.2
Unknown/Not reported	1	1.1

### Procedure

An online survey system was used to collect all of the data (Qualtrics^XM^; www.qualtrics.co.uk). Prior to the study commencing, ethical approval was obtained from a UK institutional (University College London, Institute of Education) Research Ethics Committee (REC: 1,227) and informed consent was completed for all participants.

### Design

The data from this study were derived from a larger research project. Prior to the pandemic in the UK a large-scale online survey to evaluate sleep was conducted with autistic adults (November 2019 - January 2020; Time 1). Once lockdown began in the UK, participants from this initial survey (*n* = 493), who had agreed to be contacted about future research, were invited to complete an adapted version of the original survey (including COVID-19 related questions along with selected measures from survey Time 1) first in April 2020 (Time 2) (*n* = 167), and second in June 2020 (Time 3) (*n* = 95). The response rate for participants who fully completed the survey at all three-time points was 19.3%.

### Measures

Two standardised questionnaire measures plus a range of demographic and COVID-19 related questions were included in the survey. Participants either responded on a Likert-scale or 299 answered open-ended questions, which were then categorized.

### Demographic Questionnaire

COVID-19 related questions included at Times 2 and 3 consisted of 20 items. The questions asked about whether self-isolating and/or shielding, whether experiencing symptoms, been tested or received a diagnosis, and whether anyone known to the participant has had COVID-19 and/or died as a result. In addition, questions were asked about pre-existing health conditions (“*Did you have any pre-existing mental health issues prior to COVID-19*”) and whether they had been made worse by the effect of COVID-19 if participants had reported pre-existing health conditions (“*Do you feel COVID-19 has made your pre-existing mental health issues worse”*) or whether the effect of COVID-19 had impacted on their health if no pre-existing health conditions were reported (“*Do you feel COVID-19 has impacted on your mental health causing issues where there previously was little to no problem”*).

Information was also obtained about the impact of COVID-19 on sleep and in what ways sleep has been affected (e.g., “*In what ways has your sleep been affected*”) and the impact of COVID on depression and anxiety (e.g., “*Has COVID-19 made you feel more depressed than before”*). Moreover, at Time 3, participants were additionally asked if their sleep and/or mental health had changed due to reasons not COVID-19 related and if so, what these reasons were. Finally, participants were asked if they were receiving support for a mental health issue prior to COVID-19. If so, they were asked if their support had stopped at Time 2 (“*Has your support stopped”*) and if their support had changed at Time 3 (“*Has your support changed since the last follow-up”*).

### Sleep Quality: Pittsburgh Sleep Quality Index

The Pittsburgh Sleep Quality Index (PSQI) is a retrospective self-report questionnaire which measures sleep quality over a 1-month time interval (21). It consists of 19 self-rated items and 5 additional items rated by a partner or roommate which are not included in the subscale or total score. It measures sleep disturbances across seven dimensions: (1) subjective sleep quality, (2) sleep latency, (3) sleep duration, (4) habitual sleep efficiency, (5) sleep disturbances, (6) use of sleep medication and (7) daytime dysfunction. Higher scores on the PSQI indicate worse sleep quality. The clinometric and clinical properties of PSQI demonstrate its utility in both clinical practise and research (21). This scale has previously shown good internal consistency in autistic adults (Cronbach's α = 0.68) (3). In the current study, the PSQI demonstrated good internal consistency for the seven components of the PSQI at Time 1 survey (Cronbach's α = 0.60).

### Sleep Arousal: Pre-sleep Arousal Scale

The Pre-sleep Arousal Scale (PSAS) is a 16-item self-report measure of pre-sleep arousal (22). Eight items evaluate symptoms of cognitive arousal (e.g., intrusive thoughts) and eight items evaluate symptoms of somatic arousal (e.g., sweating). Higher scores indicate higher pre-sleep arousal. The PSAS has been shown to be a useful tool for capturing the pre-sleep state and has been used as a screening measure in those with sleep disturbance (22). This scale has previously demonstrated internal consistency and stability over time, in normal sleepers, for both cognitive and somatic subscales, respectively (Cronbach's α = 0.67 and Cronbach's α = 0.84) (22). In this study, the PSAS demonstrated good internal consistency for the cognitive and somatic subscales at Time 1 survey, respectively (Cronbach's α = 0.89 and Cronbach's α = 0.81).

### Statistical Approach

The data were analysed using SPSS version 26. For COVID specific demographic data, descriptive statistics are reported. Following tests of normality conducted for the PSQI and PSAS total scores and subscales, inferential statistics were conducted to establish if age, ethnicity, or gender were significantly associated with either sleep quality (PSQI) or sleep arousal (PSAS). Subscales of the PSQI and PSAS were not normally distributed and therefore non-parametric tests reported for these outcomes. As this study included self-diagnosed autistic adults, comparisons between the self-diagnosed vs. HCP diagnosed groups were conducted for both sleep quality and pre-sleep arousal.

#### Sleep Quality and Sleep Arousal Changes Over Time

To evaluate changes in sleep quality and pre-sleep arousal a repeated measures ANOVA was conducted on the PSQI and PSAS total scores and subscales for participants who completed the survey across all three time points. When Mauchly's test was violated (<0.05) degrees of freedom were corrected, and the appropriate correction based on the test statistic is reported. Bonferroni *post-hoc* tests were conducted when the repeated measures ANOVA was significant. All results are reported for one-tailed tests. Data were available for 95 participants across the three time points.

## Results

At Time 1, no significant associations were found between age, gender, ethnicity, and sleep quality or pre-sleep arousal. Autistic adults who were self-diagnosed scored significantly higher in three areas of sleep quality compared with participants diagnosed by a HCP. These included; PSQI daytime dysfunction subscale (U = 329.00, p = 0.007, M = 2.36, SD = 0.63; M = 1.79, SD = 0.70, respectively); PSAS cognitive subscale (U = 374.50, *p* = 0.043, *M* = 30.79, SD = 7.52; *M* = 26.28, SD = 7.66, respectively); and the PSAS total score [t_(98)_ = −2.18, *p* = 0.032, *M* = 48.64, SD = 14.53; *M* = 41.74, SD = 10.27].

### COVID Related Characteristics of the Sample

In the first COVID survey (Time 2), 11.58% of respondents were self-isolating and 15.79% were shielding (*M* = 39.92 days, *SD* = 8.06). On average, respondents were living with 2.53 people in their household (including themselves). Over 8% of autistic adults reported to have experienced COVID-19 symptoms, 5.26% had been tested for COVID-19 and 1% had been diagnosed with COVID-19. Over 27% knew someone who has previously or currently suffered from COVID-19 and 12.63% knew someone who had died from COVID-19.

In the second COVID survey (Time 3), there was a much smaller number of autistic people who were self-isolating (3%), yet the number of people who were shielding remained the same (15%; *M* = 89.32 days, *SD* = 14.19). One percent were experiencing COVID-19 symptoms, 12.63% had been tested for COVID-19 and over 2% had been diagnosed with COVID-19. The percentage of participants who knew someone that had previously or currently suffered from COVID-19 increased to 36 and 16.84% knew someone who had died from COVID-19.

Table 2 summarises the consequences of the COVID-19 pandemic on mental health and sleep in autistic adults. See Supplementary Table A for additional information regarding changes in mental health support due to COVID-19 and changes in sleep and/or mental health not attributed to COVID-19.

**Table 2:** Mental health and sleep outcomes as a result of the COVID-19 pandemic in autistic adults.

	**Time 2**		**Time 3**	
	***N***	**%**	***N***	**%**
**Impact of COVID-19 on pre-existing mental health issues**				
Made pre-existing issues worse	42	50.60	30	36.14
Possibly- don't know yet	15	18.07	13	15.66
Improved pre-existing issues	14	16.87	16	19.28
Not changed pre-existing issues	12	14.46	24	28.92
**Impact of COVID-19 on sleep**				
Impacted on sleep a little	36	37.89	28	29.47
Impact on sleep a lot	29	30.53	11	11.58
Not impacted on sleep	24	25.26	46	48.42
Not sure whether impacted on sleep	6	6.32	10	10.53
**How sleep has been impacted since COVID-19**				
Waking up exhausted/tired	24	36.92	12	30.77
Not being able to get to sleep	22	33.85	13	33.30
Waking up in the night	19	29.23	8	20.51
Disrupted sleep pattern/schedule	18	27.69	8	20.51
Nightmares	12	18.46	8	20.51
Waking up too early	11	16.92	7	17.95
Poor sleep quality	11	16.92	12	30.77
Vivid dreams	10	15.38	6	15.38
Sleeping less/Insomnia worsened	10	15.38	2	5.13
Sleeping for longer	9	13.85	1	2.56
Disturbed sleep from external factors (e.g., noise)	6	9.23	4	10.26
Napping during the day	4	6.15	2	5.13
Sleeping better	3	4.62	4	10.26
**Felt more anxious than before COVID-19**				
Yes, a little	43	45.26	43	45.26
Yes, a lot	24	25.26	8	8.42
No, same as before	15	15.79	22	23.16
No, less than before	12	12.63	21	22.11
Not sure	1	1.05	1	1.05
**Felt more depressed than before COVID-19**				
Yes, a little	39	41.05	32	33.68
No, same as before	24	25.26	33	34.74
Yes, a lot	17	17.89	14	14.74
No, less than before	14	14.74	13	13.68
Not sure	1	1.05	3	3.16
**Impact of COVID-19 on mental health where previously no issues**				
No, has not caused issues	5	41.67	6	50.00
Yes, caused issues	4	33.33	1	8.33
Not sure yet	3	25.00	5	41.67

*Impact of COVID-19 on pre-existing mental health issues includes n = 83 autistic adults' who had a pre-existing mental health issues prior to COVID-19 (87.40%). How sleep has been impacted by COVID-19 includes n = 65 autistic adults at Time 2 (68.42%) and the n = 39 autistic adults at Time 3 (41.05%) who responded their sleep was impacted a lot or a little by COVID-19. Impact of COVID-19 on mental health where previously no issues includes n = 12 autistic adults' autistic adults (12.63%) who did not have any pre-existing mental health issues prior to COVID-19*.

### Changes in Sleep Quality

Table 3 displays the means and standard deviations for each sleep quality outcome across time. Several of the PSQI scores were significant across the three time points. The PSQI total scores indicated that overall sleep improved [F_(2, 172)_ = 8.10, *p* < 0.001]. Also significant improvements were found on the PSQI component scores such as sleep latency [F_(2, 176)_ = 4.42, *p* = 0.013], sleep duration [F_(2, 184)_ = 6.27, *p* = 0.002], sleep efficiency [F_(2, 178)_ = 5.01, *p* = 0.008] and daytime dysfunction [F_(2, 188)_ = 10.02, *p* < 0.001] demonstrated significant differences across the three time points. PSQI component scores of subjective sleep quality [F_(2, 188)_ = 0.64, *p* = 0.527], sleep disturbance [F_(2, 188)_ = 2.24, *p* = 0.109] and sleep medication [F_(1.84, 172.54)_ = 1.46, *p* = 0.236] were not significant across the three time points. Further analysis, using Bonferroni corrected comparisons, indicated differences between the three time points. PSQI total score (*p* < 0.001), and component scores of sleep latency (*p* = 0.025), sleep duration (*p* = 0.005), and daytime dysfunction (*p* = 0.001) were all lower at Times 2 and 3 than Time 1 (*p* values represent differences between Time 1 and Time 3). PSQI component score of sleep efficiency was lower at Time 3 than Time 1 (*p* = 0.015), however no difference was found between Times 1 and 2 (*p* = 1.00).

**Table 3:** Mean scores of sleep quality and pre-sleep arousal at Times 1, 2, and 3.

		**Time 1**		**Time 2**		**Time 3**	
**Measures**	**Subscales**	***M***	***SE***	***M***	***SE***	***M***	***SE***
PSQI	Subjective sleep quality	1.82	0.67	1.77	0.74	1.74	0.84
	Sleep latency	2.18	0.11	1.96	0.11	1.94	0.09
	Sleep duration	1.08	0.11	0.83	0.11	0.74	0.11
	Sleep efficiency	1.44	0.13	1.39	0.13	1.08	0.12
	Sleep disturbance	1.64	0.06	1.52	0.06	1.62	0.06
	Sleep medication	0.73	0.12	0.85	0.12	0.91	0.13
	Daytime dysfunction	1.87	0.07	1.58	0.08	1.60	0.08
PSQI total		10.69	0.39	9.80	0.44	9.44	0.42
PSAS	Somatic arousal	15.89	0.59	15.93	0.65	16.16	0.64
	Cognitive arousal	26.94	0.81	25.24	0.75	25.19	0.84
PSAS total		42.83	1.16	41.17	1.20	41.35	1.32

*PSQI, pittsburgh sleep quality index; PSAS, pre-sleep arousal scale*.

### Sleep Arousal Changes

PSAS total score [F_(1.76, 163.58)_ = 2.18, *p* = 0.123] and the PSAS somatic subscale scores [F_(1.72, 160.05)_ = 0.18, *p* = 0.801] were not significant across the three time points. However, scores on the cognitive arousal subscale demonstrated a significant change across the three time points [F_(1.88, 174.55)_ = 5.61, *p* = 0.005]. Bonferroni corrected comparisons indicated PSAS cognitive arousal score was significantly lower at Time 2 compared to Time 1 (*p* = 0.031) and at Time 3 compared to Time 1 (*p* = 0.016).

## Discussion

The findings from the current study suggest several sleep components improved during lockdown. These included reduced sleep latency (time taken to fall asleep), longer sleep duration, improved sleep efficiency, sleep quality as well as improved daytime functioning. Most of the sleep components gradually reduced over the three time points, with the highest values at Time 1 (pre-lockdown) and gradually reducing, suggesting that the initial lockdown (Time 2) had a positive impact on autistic adults sleep and pre-sleep arousal overall, and this was maintained at Time 3. Pre-sleep cognitive arousal scores were found to decrease compared to pre-lockdown, meaning cognitive arousal improved. However, only three participants (4.62%) reported they were sleeping better at Time 2, and 4 (10.26%) at Time 3. Approximately 65% of participants reported that they felt their sleep had been impacted since the COVID-19 pandemic at Time 2, with the most common reasons waking up exhausted (36.92%), not being able to get to sleep (33.85%), waking up in the night (29.23%), disrupted sleep pattern (27.69%), and nightmares (18.46%), suggesting there were also negative changes to sleep for autistic adults in this self-reported data. It is possible that these self-reported sleep problems from the demographic questions were present prior to the pandemic and participants did not report only new symptoms that have developed since the pandemic started, in addition these symptoms may have been exacerbated since the pandemic began. It should be noted that the PSQI positive changes reported are in the context of a population who already had high levels of sleep problems, reflected in the Time 1 data. The PSQI has a sensitivity of 89.6% and specificity of 86.5% for identifying cases with sleep disorder, using a cut-off score of 5 (23). In our sample, the mean total PSQI for Time 1 was 10.69 improving to 9.44 in Time 3 demonstrating this sample is above the clinical cut off across all three time points, which is likely reflected in the demographic question responses.

As this study included autistic adults who self-reported their diagnosis and those who had reported receiving a HCP diagnosis, these two groups were compared. Participants who had a self-reported diagnosis scored higher on the PSQI daytime dysfunction subscale, the PSAS cognitive subscale, and PSAS total scores. Evidence has suggested that following a diagnosis of ASD, anxiety reduces in autistic adults, it is also possible that anxiety is related to camouflaging practises often seen in self-diagnosed individuals (24). This finding could also reflect a lack of healthcare support provided to autistic individuals who have not received a diagnosis leading to higher anxiety outcomes (25).

Improvements in the PSQI sleep component scores may be linked to commonly reported circadian rhythm disorders in autistic adults and the removal of the need to adhere to the social structure of a typical day, may have allowed adults to sleep according to their natural cycles, thus overall improving sleep. Tani et al., (26) reported that autistic adults, when compared with typically developing controls, were more often diagnosed with insomnia, and it was suggested that neuropsychiatric divergences characteristic of autistic adults predispose autistic adults to insomnia. The reduction in pre-sleep cognitive arousal in this study is in keeping with decreased sleep latency scores in the PSQI. In line with Tani et al., (26), the current findings suggest that environmental factor/s play a strong contributing role in sleep patterns of autistic adults. The pandemic has led to a range of societal adjustments such as working from home, restrictions in commuting, online shopping, socialising, medical appointments and use of telehealth as a mode of gaining advice/treatment. Some of these changes are arguably beneficial for autistic adults and allow for much needed long-term adjustments in for instance, employment and societal norms, contributing to a more inclusive autism-friendly society. For example, in children and adolescents, home-schooling allows for a greater opportunity to obtain sufficient sleep due to flexible waking times and respite from potential bullying stressors (5).

Moreover, some studies have reported positive effects of the COVID-19 pandemic on the mental health of autistic adults (8, 15, 27, 28). Positive outcomes and coping strategies reported by autistic adults include being able to use extra time to pursue hobbies, establishing new routines, spending more time with family, keeping busy engaging in chores, adapting to virtual social connexion, spending time in nature and learning at a comfortable pace (15, 27). Spending time outdoors “in nature” has more generally been promoted as an effective adaptive strategy during the pandemic (29). Furthermore, autistic adults have demonstrated great resilience in day care settings where no negative impacts on behaviour were uncovered as a result of the pandemic (30, 31). These positive outcomes may be a result of decreased environmental requirements including social stress and sensory demands- which often require ample attention and energy expenditure (28). All of these changes could have contributed to a decrease in cognitive arousal scores.

However, of the autistic adults who reported negative changes to their sleep, these findings may also suggest that mental health difficulties may be playing a role in reports of waking up exhausted and nightmares. In our sample, at Time 2 at the start of the pandemic, 50.60% of participants reported the impact of COVID-19 worsening pre-existing mental health conditions; 70.52% reported feeling more anxious; and 58.94% reported feeling more depressed than before than pandemic. By Time 3, 36.14% of participants reported the impact of COVID-19 worsening pre-existing mental health conditions; 53.68% reported feeling more anxious; and 48.6% reported feeling more depressed than before the pandemic. For example, Oomen et al., (8) found an increase in depression and anxiety symptoms in response to the pandemic for autistic adults, participants also showed a greater interest in worries about pets, work, access to medication and food compared to non-autistic peers. Therefore, although there may have been some positive impact on sleep in autistic adults, the overall impact of the pandemic on mental health and sleep, and complex interactions between these two factors, requires further investigation. In addition, it is important to consider that prior to the pandemic, many of the autistic adults in this sample already experienced high levels of sleep problems.

## Limitations

There are several limitations to consider for this study. The timeframe in which the data was collected was during the first lockdown period in the UK. It is not known how these outcomes may have changed in prolonged periods of lockdown, specifically if this sleep benefit has been maintained throughout subsequent lockdowns. Although daily schedule remains similar e.g., working from home, virtual socialising, flexible working patterns, there now may be additional worries such as job prospects, money, when/if normality will return. In addition, we did not collect employment status for Times 2 and 3 for comparison, which would have helped to contextualise additional changes which may have contributed to the results. The response rate for participants who fully completed the survey at three time points was relatively low at 19.3%, however, similar studies for attrition rate comparison could not be found. Therefore, it should be considered that this survey may not be fully representative of all the autistic population. It is possible that many autistic adults who have been significantly negatively impacted, were not willing to participate in the survey. In addition, we did not have a comparison group to compare the results to. For example, the inclusion of a typically developing control group or a patient control group (e.g., psychosis patient control group). This would have enabled us to better support our hypotheses that mental health difficulties have a specific influence on sleep difficulties in autistic adults. Moreover, although we included autistic adults who were diagnosed by a HCP and those who were self-diagnosed, autistic adults comprise of a wide range of individuals with diverse clinical representations. Relatedly, given the demands of completing an online survey, it is likely our autistic adult population excludes those autistic adults on the lower-functioning part of the autism spectrum. Future studies should endeavour to overcome this criticism by encouraging carers to help with the survey in these instances. A final limitation relates to the issue that the online surveys did not provide the opportunity to check or confirm the presence of a diagnosis of autism. Therefore, as we were reliant on self-report we cannot confirm all participants self-disclosed accurately. However, during the Time 1 survey we did ask participants to provide information on their diagnosis (e.g., age of diagnosis, professional who gave the diagnosis and month and year of the diagnosis) to potentially limit this issue. A recent study by McDonald (25) provided a comparison between autistic adults that are self-diagnosed vs. diagnosed by a HCP, the study found the inclusion of self-reported autistic adults in online surveys likely represents the “lost generation” of autistic adults, such as older females. In our sample there are three times more females than males included aged between 18 and 65 years old. This demographic is likely a reflection of our recruitment strategy and collaboration with UK charitable organisations which include and represent both autistic adults with a HCP diagnosis and those who identify as self-diagnosed.

## Conclusions and Future Research Direction

In the general population, delayed sleep onset and nocturnal sleep fragmentation have been reported to lead to emotional distress, daytime fatigue and loss of productivity (32). Furthermore, autistic adults who experienced sleep disturbances are more likely to be unemployed compared to autistic adults without sleep problems (33). These findings suggest that sleep problems are an important predictor of later quality of life for autistic adults, offering an important treatment avenue for improving quality of life in this population (34). In terms of creating a more autism-inclusive society, societal changes which have been reportedly beneficial in autisitc adults as a result of the pandemic include: telehealth opportunities for care and therapies, online opportunities for conferences and events, remote learning courses, creating calm and minimised emotionally distressing healthcare environments (13, 14, 27). Cassidy et al., (14) goes on to further argue now is the best time to figure out how to best include autistic people in society. Taken together, these long-term changes could result in more flexible and empathic working environments, improved access to resources and diverse health and treatment invention options, ultimately leading to a more autism inclusive future after the pandemic. Although the findings in this study are limited to the UK, imposed restrictions and solutions were global, and therefore these findings may provide some evidence across countries.

## Data Availability Statement

The raw data supporting the conclusions of this article will be made available by the authors, without undue reservation, upon request.

## Ethics Statement

The studies involving human participants were reviewed and approved by University College London, Institute of Education. The patients/participants provided their written informed consent to participate in this study.

## Author Contributions

EH, ES, and DD contributed to conception and design of the study. ES organised the database and wrote sections of the manuscript. EH and ES performed the statistical analysis. EH wrote the first draft of the manuscript. All authors contributed to the article and approved the submitted version.

## Funding

This work was supported by the John and Lorna Wing Foundation. The funders had no involvement in study design, data collection, data analysis, interpretation, writing or decision to submit this journal article for publication.

## Conflict of Interest

The authors declare that the research was conducted in the absence of any commercial or financial relationships that could be construed as a potential conflict of interest.

## Publisher's Note

All claims expressed in this article are solely those of the authors and do not necessarily represent those of their affiliated organizations, or those of the publisher, the editors and the reviewers. Any product that may be evaluated in this article, or claim that may be made by its manufacturer, is not guaranteed or endorsed by the publisher.
