# Supplementary material for: The Impact of COVID-19 on Sleep in Autistic Adults: Longitudinal Comparisons pre and During Lockdown
Source: Front Psychiatry. 2021 Sep 21;12:708339. doi: 10.3389/fpsyt.2021.708339 (PMC8490657; doi:10.3389/fpsyt.2021.708339)
Supplement: Supplementary file 1 [file Table_1.docx]

**Supplementary material**

**Table A.** Support-related outcomes as a result of the COVID-19 pandemic and changes in sleep and mental health not attributed to COVID-19 in autistic adults.

|  | *COVID-19 follow up one* | | *COVID-19 follow up two* | |
| --- | --- | --- | --- | --- |
|  | *N* | *%* | *N* | *%* |
| *Impact of COVID-19 on support: both current and seeking support*  Has your support stopped since COVID-19  No, offered online/phone alternative and accepted  Yes but feel ok  No, continued  Yes, made things worse  Yes, offered online/phone alternative and declined  Other (e.g. receiving limited support initially)  Has your support changed since the follow up one (April-May)  No, same as before  Yes, support now stopped  Yes, support moved online  Yes, change in circumstance (e.g., new therapist)  Yes, receiving additional support  Yes, support less frequent  Yes, support for frequent  Yes, support back in person Unsure  Sought support from mental health services since COVID-19  No, know they won’t help  No, have not felt the need  Yes, they helped  Yes, but they didn’t help  No, already supported enough  No, don’t have the energy  No, do not have time  No, lack of resources (e.g., financial)  Unknown/Not reported  *Sleep and mental health changes not attributed to COVID-19*  Sleep or mental health changed for reasons not COVID-19 related  Secondary effects of COVID-19  Unrelated health conditions  Experienced adverse life event (e.g., death of loved one)  Change in medication or non-medication treatment  Experienced a non-adverse life event (e.g., new job)  External environment (e.g. noise, heat)  Other ongoing global events (e.g., BLM movement)  Worried about future (e.g. career)  Unsure | 25  10  7  3  1  1  -  -  -  -  -  -  -  -  -    36 23  13 11  10  1  1 - -     -  -  -  -  -  -  -  -  - | 53.19  21.28  14.89  6.38  2.38  2.38  -  -  -  -  -  -  -  -  -  37.89 24.21 13.68  11.58  10.53  1.05 1.05 - -  -  -  -  -  -  -  -  -  - | - - - -  -  -  22  5  4  4  3  1  1  1  1  28  35  15  7  6  1  1  1  1  12 9 7 6 3 3 2 1 1 | - - - -  -  -  52.38  11.90  9.52  9.52  7.14  2.38  2.38  2.38  2.38  29.47  36.84  15.79  7.37  6.32  1.05  1.05  1.05  1.05  30.77 23.08 17.95 15.38 7.69 7.69 5.13 2.56  2.56 |

*Notes.* Has your support stopped or changed since COVID-19 includes *n* = 47 (49.47%) in follow up one and *n* = 42 (44.21%) in follow up two who were receiving support for a pre-existing mental health issue prior to COVID-19. Whether sleep or mental health has changed for reasons not COVID-19 related includes *n* = 39 in follow up two who indicated their mental health had changed for reasons not COVID-19 related (41.05%). BLM = Black Lives Matter.
